# Stretching and potentiation for performance optimization: effects on upper limbs in competitive swimmers

**DOI:** 10.1007/s00421-026-06133-9

**Published:** 2026-01-16

**Authors:** Francisco Cuenca-Fernández, Andreas Konrad, Markus Tilp

**Affiliations:** 1 Department of Sports and Computer Sciences, Universidad Pablo de Olavide, Seville, Spain; 2 Aquatics Laboratory, Department of Physical Education and Sports, Faculty of Sport Sciences, University of Granda, Granada, Spain; 3 Department of Human Movement Science, Institute of Human Movement Science, Sport and Health, University of Graz, Mozartgasse 14, 8010 Graz, Austria

**Keywords:** Upper-limbs biomechanics, PAPE, Neuromuscular performance, Warm-up protocols, Overhead athletes

## Abstract

This study examined the acute effects of combining short-duration static stretching with resistance-based activation, designed to induce post-activation performance enhancement (PAPE), on upper-limb muscle mechanics and neuromuscular performance. Fourteen competitive swimmers (7 male, 7 female) completed three randomized warm-up protocols: (1) general aerobic activity (CON), (2) Aerobic activity + 2 × 30 s of static stretching for latissimus dorsi and pectoralis major (SS), and (3) Aerobic activity + static stretching + 3 × 5 reps of resistance-band pull-overs (SS/PAPE). Shoulder extension range of motion (ROM), pectoralis- and latissimus dorsi-passive muscle stiffness and elasticity as well as isometric/dynamic torque, rate of force development (RFD), and power (isokinetic dynamometer) were assessed pre- and 10 min post-intervention. Results showed significant Time × Protocol interactions for ROM, maximal torque, RFD, and average torque. SS/PAPE elicited the largest ROM gain and improvements in dynamic performance, with significant increases in average torque and RFD, while SS led to modest ROM increases but small and non-significant declines in average torque and RFD. Passive muscle stiffness and elasticity exhibited non-significant changes in all groups. Although relative changes were comparable across sexes, males consistently outperformed females in torque and RFD, while females presented higher ROM than males. These findings demonstrate that integrating brief static stretching with sport-specific, high-intensity upper-limb activation can acutely enhance flexibility and explosive force generation without impairing performance. The Stretching + PAPE approach offers a practical, time-efficient warm-up strategy for overhead athletes, particularly in contexts where rapid force production at extended joint positions is essential.

**Supplementary Information:**

The online version contains supplementary material available at 10.1007/s00421-026-06133-9.

## Introduction

Athletes and coaches continually seek effective strategies to optimize performance, particularly through competition preparation methods. Among these, warm-up routines are widely used across sports and typically incorporate general aerobic activity followed by more task-specific, high-intensity exercises. These routines have consistently been shown to acutely enhance performance by increasing muscle temperature, elevating metabolic rate, and improving neuromuscular readiness (Bishop 2003; Boullosa 2021). Equally, flexibility is a well-established factor commonly integrated into warm-ups, as increased range of motion (ROM) is associated with improved movement efficiency and injury risk reduction in activities requiring large joint excursions, such as swimming, dance, and gymnastics (Behm et al. 2020; Lima et al. 2019). As such, pre-performance routines frequently include stretching and mobilization exercises alongside dynamic warm-up components (Fradkin et al. 2010; McCrary et al. 2015).

While the acute physiological benefits of warm-up are well-documented (Bishop 2003; Blazevich and Babault 2019), the mechanistic underpinnings (e.g., ROM, muscle stiffness, rate force development [RFD]) remain underexplored, particularly in the upper limbs (Finlay et al. 2021). Similarly, although static stretching is known to increase range of motion (ROM) through enhanced stretch tolerance and reduced passive muscle stiffness (Kay and Blazevich 2012; Konrad and Tilp 2014, 2020), its duration and application are critical. For instance, while prolonged static stretching (> 60 s per muscle group) can reduce maximal voluntary contraction (MVC) (Behm et al. 2021, 2020; Konrad et al. 2019), shorter durations (< 60 s) tend not to impair strength or power, and have been shown to maintain ROM improvements for up to 30–40 min (Konrad and Tilp 2020; Sato et al. 2020). Additionally, dynamic activities immediately following stretching can mitigate small reductions in force, restoring or maintaining performance (Blazevich et al. 2018; Reid et al. 2018; Samson et al. 2012). In this regard, recent insights on Post-Activation Performance Enhancement (PAPE) have expanded the understanding of warm-up effects beyond the classical myosin-phosphorylation–based Post-Activation Potentiation (PAP) framework (dissipates rapidly ~ 30 s) (Cuenca-Fernández et al. 2017; Prieske et al. 2020). Rather than specific fast-dissipating neural mechanisms (i.e., PAP), PAPE refers to performance improvements developing in the minutes after high-intensity activity and is attributed to increases in muscle temperature, fluid shifts, metabolic activation, and general neuromuscular readiness, all of which can sustain performance enhancements across diverse sport-specific tasks (Blazevich and Babault 2019; Boullosa 2021). These effects typically peak 5–10 min post-exercise (Xu et al. 2025) and are influenced by factors such as training background, muscle fiber type composition, contraction type, and the recovery interval (Seitz and Haff 2016; Wilson et al. 2013; Cuenca‐Fernández et al. 2024). Although most PAPE research focuses on the lower limbs, similar temperature- and activation-related mechanisms have been reported in the upper body during tasks such as throwing or resisted pulling (Mascarin et al. 2015; Gelen et al. 2012; Cuenca-Fernández et al. 2022).

Interestingly, the combination of stretching followed by high-resistance or dynamic exercises presents a promising method to obtain both ROM and PAPE-related benefits. Short-duration static stretching (< 60 s) primarily increases joint ROM through enhanced stretch tolerance and reductions in passive resistance (i.e., muscle stiffness), whereas acute decreases in voluntary force production are typically associated only with longer stretching durations (~ 120 s), which are uncommon in sports settings, or with protocols sufficient to induce measurable changes in tendon stiffness (Avela et al. 2004; Behm et al. 2020; Kubo et al. 2002; Konrad and Tilp 2020). In contrast, PAPE-oriented activities, particularly those involving high or submaximal loads, could improve motor unit recruitment efficiency and synchrony (Boullosa 2021; Seitz & Haff 2016), factors associated with short-term improvements in force production and power output (i.e., RFD) (Blazevich and Babault 2019; Boullosa 2021). Because passive mechanical effects of stretching and the physiological responses associated with PAPE evolve on different time courses (Blazevich et al. 2018; Cè et al. 2008; Mascarin et al. 2015), sequencing a brief static stretch with an immediate dynamic or resisted activation may preserve ROM gains while maintaining or restoring performance (Konrad et al. 2019; Prieske et al. 2020). These combined approaches have demonstrated efficacy in the lower limbs (Behm et al. 2020). However, findings remain inconsistent on the upper limbs, likely due to variations in stretch duration, participant training level, or the sequence and intensity of conditioning activities (Haag et al. 2010; Leone et al. 2014; Torres et al. 2008).

Given the functional complexity of overhead actions and the vulnerability of the shoulder to mobility-stability imbalances, optimizing warm-up strategies for the upper body is of particular relevance in sports involving throwing, swimming, or overhead lifting (Laudner and Sipes 2009; Møller et al. 2017; Umehara et al. 2018). Evidence in this area is mixed: some studies have reported acute reductions in maximal strength and power following prolonged static stretching (≥ 60 s per muscle group) of the shoulder and elbow flexors (Herda et al. 2008; Leone et al. 2014), whereas others have observed increased throwing velocity, improved shoulder ROM, or maintained force output in overhead athletes when shorter-stretching durations or dynamic protocols were used (Gelen et al. 2012; Ghareeb et al. 2017; Mascarin et al. 2015). Muscles such as the latissimus dorsi and pectoralis major play critical roles in force production during overhead tasks, yet their acute mechanical responses to combined stretching and warm-up interventions remain poorly understood. Fortunately, recent advances in ultrasound imaging and myotonometry now allow detailed, non-invasive assessment of muscle architecture and passive stiffness, providing new opportunities to investigate how different warm-up strategies influence upper-limb musculature also in the different sexes (Agyapong-Badu et al. 2016; Freitas et al. 2018).

To date, no study has directly examined the acute mechanical and functional responses of the upper limbs to a conditioning protocol combining static stretching and dynamic-resisted warm-up activities. Therefore, the aim of this study was to to evaluate the acute effects of such a protocol on upper-limb muscle mechanics and performance in a group of competitive swimmers. Given that ROM increases from stretching can persist for 30–40 min (Behm et al. 2023), and that PAPE effects typically peak within 5–10 min post-activity (Xu et al. 2025), we hypothesised that a short static stretch (2 × 30 s) would increase ROM primarily through enhanced stretch tolerance while the subsequent PAPE-oriented activation is expected to increase muscle readiness within a 5–10 min window, potentially counteracting any transient performance reductions following stretching. Thus, the combination is expected to be synergistic (increases in ROM and in neuromuscular readiness) rather than purely antagonistic.

## Methods

### Experimental approach to the problem

**Fig. 1:**
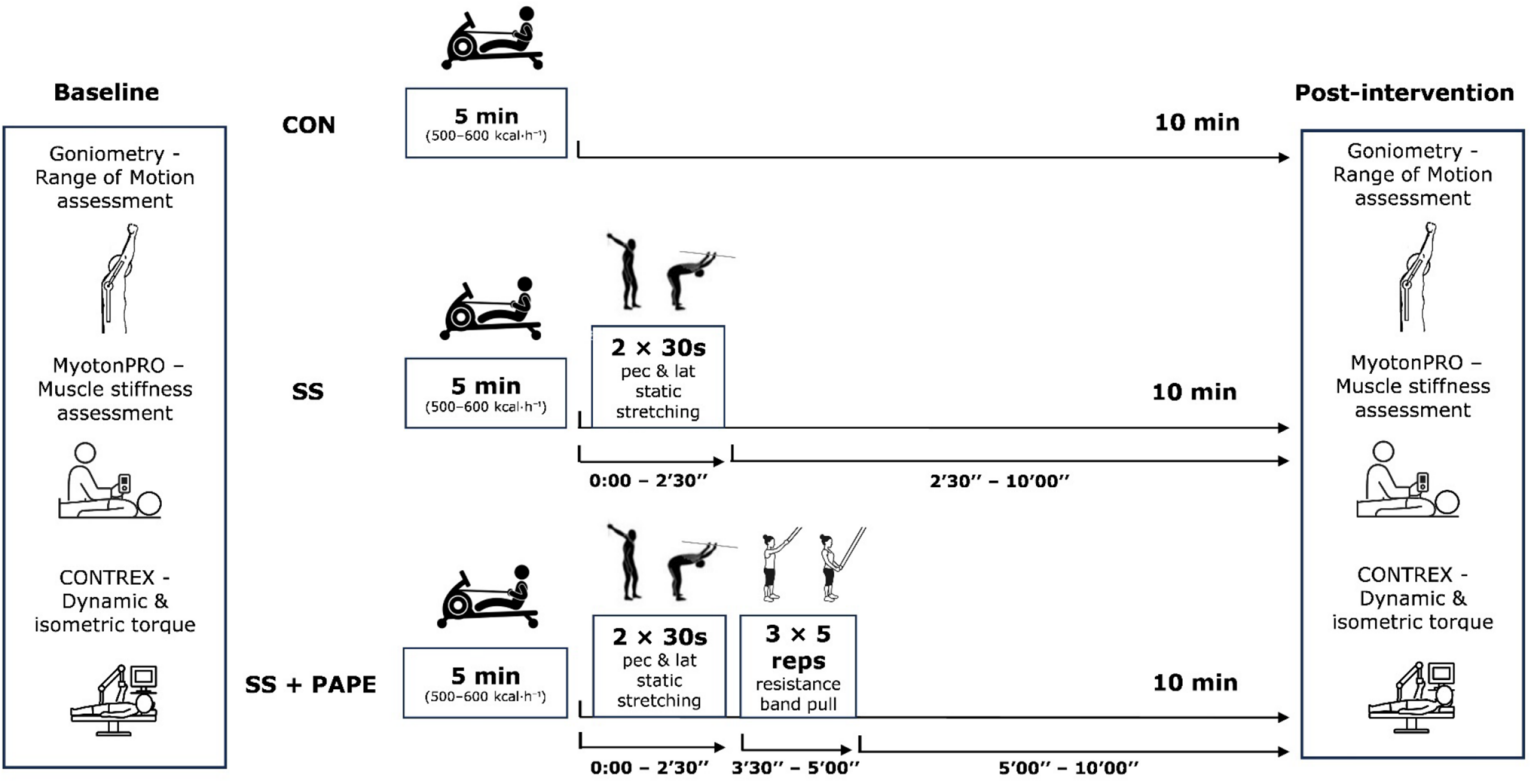
Schematic schedule including a presentation of the interventions (CON: Control condition; SS: Static Stretching, and; SS + PAPE: Static stretching followed by elastic bands repetitions)

A repeated measures design was employed to test the pre-post effects in the upper limbs muscle mechanics and function of shoulder extension after three different warm-up conditions. Primary outcomes included shoulder extension ROM, passive muscle stiffness of latissimus dorsi and pectoralis major, and dynamic average torque (isokinetic 180°·s^−1^); while secondary outcomes included passive muscle elasticity, dynamic peak torque, work, and power, and isometric MVC at three angles (150°, 90° and 35°) (Fig. 1). Test variables were assessed (2 min) before and (10 min) after the warm-up exercises to assess potential PAPE responses (Xu et al. 2025). All participants completed three laboratory dryland sessions in randomized order using a computer-generated block randomization scheme (1:1:1), with a minimum 48-h washout. The participants underwent three different warm-up protocols: Control (CON): A general warm-up condition including 5 min of aerobic activity was conducted and acted as control; Static stretching (SS): a static stretching technique was added to the general activity; Static stretching and PAPE (SS/PAPE): resistance-dynamic exercises were added to the general warm-up and stretching protocol. Assessors remained blinded to session order. Testing was performed at the same time of day for each participant to minimize circadian variation.

### Participants

A priori power analysis (G*Power; Faul et al. (2007)) for a 2 (time: pre/post) × 3 (protocol) repeated-measures ANOVA was performed. We assumed a moderate-to-large effect (ηp^2^ ≈ 0.24; equivalent to f ≈ 0.5) based on previous studies that investigated the effect of 2 × 30 s of SS on the lower extremities ROM (mean change = 12%, SD = 16%, d = 0.73 = moderate to large effect) (Konrad and Tilp 2020); and the effect of 2 × 5 reps of resisted band pull on upper-limbs peak thrust (mean change = 13.37%, SD = 15%, d = 0,73 = moderate to large effect) (Barbosa et al. 2020). With α = 0.05, correlation among repeated measures = 0.50, and desired power = 0.80, the minimum sample was n = 10 (n = 12 for 90% power). To allow for two pre-specified primary outcomes (shoulder ROM and average dynamic torque/dRFD) and potential attrition, we recruited 14 swimmers for the study. This consisted of seven men (19.1 ± 2.6 years; 78.4 ± 5.9 kg; 181.1 ± 4.4 cm; 100 m freestyle best pace: 57.20 ± 1.89 s) and seven women (19.7 ± 1.9 years; 71.4 ± 3.7 kg; 174.8 ± 4.2 cm; 100 m freestyle best pace: 61.79 ± 2.41 s). All participants provided written informed consent (with parental/guardian consent for those under 18). The sample comprised elite, competitive, or highly trained amateurs (≥ 10 h·wk^−1^ combined swim + gym; ≥ 5 years’ experience), which were classified as performance Level 4, according to Ruiz-Navarro et al. (2022). Exclusion criteria were: (1) ≤ 16 years; (2) injury, surgery, or disease in the past 6 months; (3) current use of drugs or performance-related supplements; (4) unwillingness to comply with measurement requirements or scheduling. The study was approved by the local ethics committee (GZ. 39/56/63 ex 2024/25) and conducted in accordance with the Declaration of Helsinki.

### Warm-up exercises

- Aerobic activity: All sessions began with a standardized 5-min aerobic warm-up on a rowing ergometer, during which exercise intensity was regulated via the machine’s displayed caloric expenditure. Swimmers maintained a workload of approximately 500 kcal·h^−1^ in the initial four minutes, increasing to 500–600 kcal·h^−1^ in the final minute, offering an effective aerobic activation that elevates cardiovascular and muscular readiness without inducing undue fatigue (Cardoso et al. 2024).
- Static stretching: Consisted of a slow passive maneuver until a maximum ROM was attained, in a position that subjects reported feeling of maximal stretch but no pain. The exercise position to stretch the latissimus pectoralis major consisted of arm-extension including contralateral rotation of the trunk (Leone et al. 2014; Mascarin et al. 2015) and body bend with arms holding on a bar to stretch the latissimus dorsi (Asayama et al. 2021). These positions were held for 30 s and performed twice with less than 30 s of rest between sets.
- Resistance exercise: To induce PAPE responses, 3 × 5 repetitions of bilateral resistance band pull were performed one minute after the stretching, with < 1 min of rest between sets. These protocol were chosen due to its capacity of impact the arm-pull (Barbosa et al. 2020). Band resistance was individually adjusted so that participants could complete the target repetitions at a high but submaximal effort, with a margin of 1–2 reps in reserve (RIR), (i.e., 5 reps from 6 or 7) (González-Badillo and Sánchez-Medina 2010). This prescription ensured participants worked close to their maximum capacity while avoiding excessive fatigue, which is consistent with RIR-based training load control.

A fixed 10-min recovery period followed the end of each aerobic warm-up (Fig. 1), to align with known PAPE time courses (Xu et al. 2025). All procedures were performed in an indoor exercise physiology laboratory maintained at ~ 23 °C, with controlled ventilation and stable ambient humidity. The adjacent strength-training area used for the activation exercises was embedded within the same building and shared the same environmental control. Participants wore standard sports clothing (shorts and T-shirt or tracksuit) for the warm-up, activation, and dynamometer testing. For muscle stiffness measurements, swimmers briefly exposed the shoulder region (swimsuit or sports top) to allow accurate probe placement and were then instructed to redress during all rest periods to minimise cooling. No participant was exposed to outdoor conditions at any point during the testing session.

### Data collection for the muscle mechanics

#### Range of motion measures (ROM)

To investigate ROM, participants were assessed using an adapted manual goniometer mounted on a wall side. Facing the wall, they held a horizontal stick attached to a goniometer fixed on the wall side and began in a neutral arm-extended position at 0°. From this starting point, they elevated their arms forward and upward to 180°, aligning with full overhead extension. Without changing grip, participants then continued the movement into maximum backward extension, allowing assessment of their full passive overhead and posterior shoulder mobility. End ROM was determined by joint end feel, allowing for measurement in a consistent manner (van den Hoorn et al. 2025; Norkin and White 2016). This setup minimized variations in positioning, ensured consistent alignment with the measurement axis, and reduced reliance on muscular effort, focusing instead on the joint’s passive capacity. The difference between the neutral arm-extended position and the maximum stretching position was defined as the arm-extended ROM (Konrad and Tilp 2020). Passive ROM goniometric assessment has been shown to be the gold standard and reliable when performed by the same tester (Hayes et al. 2001). With a single examiner using this method, a rotational measurement error of ± 3° has been reported (Kolber and Hanney 2012).

#### Muscle stiffness and elasticity

To investigate possible structural mechanisms such as changes in muscle stiffness, a MyotonPRO® (Myoton AS, Tallinn, Estonia) device was used. The MyotonPRO® is a portable, non-invasive device designed to objectively assess the mechanical and viscoelastic properties of soft tissues, including muscles, tendons, and skin (Lettner et al. 2024). It operates by delivering a brief mechanical impulse through a small probe placed on the skin, which induces oscillations in the underlying tissue. These oscillations were automatically analyzed by the device to calculate *dynamic stiffness* (S), expressed in newtons per meter (Nm^−1^), indicating the tissue’s resistance to deformation, and; *elasticity* (D), measured by the logarithmic decrement, a dimensionless value that reflects the tissue’s ability to return to its original shape. The MyotonPRO® has been validated in various clinical and research context in sports science. Numerous studies have confirmed the device’s reliability and accuracy in quantifying the biomechanical characteristics of soft tissues, with excellent intra- and inter-rater reliability (ICC > 0.80) across different muscles and populations (Agyapong-Badu et al. 2016; Lettner et al. 2024; Bailey et al. 2013). The MyotonPRO® was applied on the muscle bellies of the pectoralis major, latissimus dorsi. Measurements were taken on the pectoralis major (clavicular head) and the latissimus dorsi. For the pectoralis major, the probe was positioned approximately 1 cm inferior to the clavicle at the midpoint of the clavicular head, over the muscle belly, as previously described in upper-limb stiffness protocols (Leonardis et al. 2019; Taş et al. 2023). For the latissimus dorsi, the measurement site was standardized at the midpoint of the lumbar–costal region, corresponding to the intersection of the vertical line from the spinous process of the eighth thoracic vertebra (Th8) and the horizontal line connecting the spinous process of the first lumbar vertebra (L1) to the crest of the lesser tubercle (Asayama et al. 2021). Prior to measurement, the probe was placed perpendicular to the skin surface with light contact force, and participants remained relaxed in a prone position to minimize active muscle tension.

#### Dynamometry measures

**Fig. 2:**
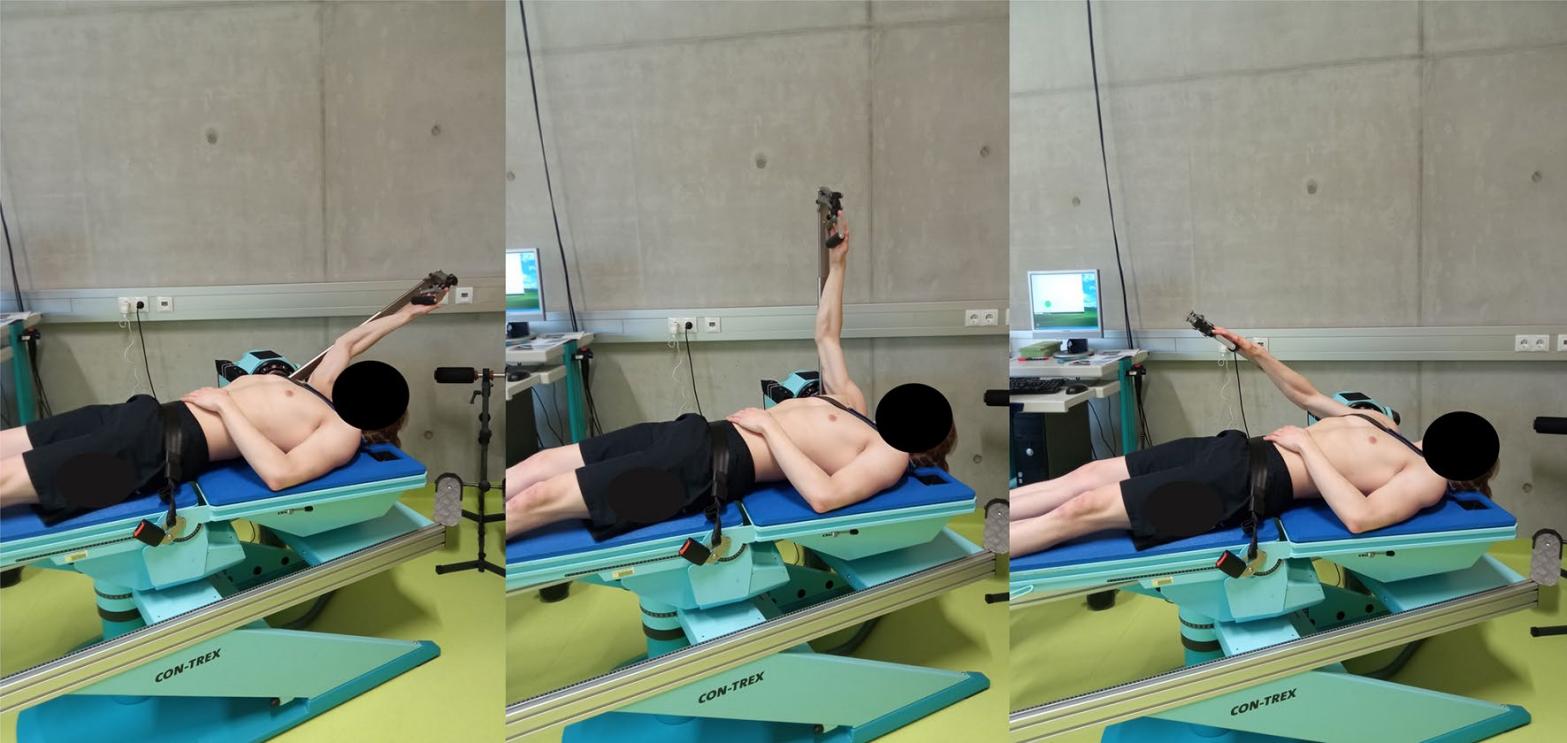
Maximal voluntary contractions (MVC) of shoulder flexions at three shoulder joint angles, 150°, 90°, and 35° degrees

Dynamic and isometric shoulder extension performance was assessed using an isokinetic dynamometer (CON-TREX MJ, CMV AG, Duebendorf, Switzerland) (Chodock et al. 2020) using its upper limb measurement set-up as previously reported with swimmers (Knihs et al. 2025). Participants were positioned supine with the tested arm extended and the shoulder joint aligned to the dynamometer’s axis of rotation. The trunk was stabilized using two oblique straps to minimize compensatory movements. For dynamic assessments, concentric shoulder extension trials were performed at an angular velocity of 180°·s^−1^ across a full individualized range of motion (from 180° flexion to 0°), allowing quantification of *peak torque* (Nm), *time to peak torque* (s), *dynamic rate of force development* (dRFD, Nm·s^−1^), *average torque* (Nm), *work* (J), and *power* (W). Each participant completed five maximal efforts and the average values were used for analysis. For isometric testing, maximal voluntary contractions (MVC) of shoulder flexions with an extended arm were recorded at three shoulder joint angles, 150°, 90°, and 35° of flexion (Fig. 2), representing long, mid, and short muscle lengths, respectively, and in accordance with the “Entry and Catch”, “Pull” and “Push” phases of the arm-stroke swimming motions (Chollet et al. 2000). At each angle, participants performed 2-s MVCs, with a 30 s rest between trials to avoid any fatigue (Konrad and Tilp 2020; Konrad et al. 2017). *Isometric RFD (iRFD)* was also calculated over the initial 200 ms of contraction, and *time to peak torque* was determined for each trial. All measurements were preceded by a standardized familiarization session to minimize learning effects. Visual feedback and verbal encouragement were provided to maximize effort. This approach enabled comprehensive assessment of both force-generating capacity and explosive performance under dynamic and static conditions, offering complementary insight into neuromuscular function in response to the warm-up protocols.

### Statistical analysis

Descriptive statistics were expressed as the mean ± standard deviation (SD). Measurement reliability was assessed using intraclass correlation coefficients (ICC), the standard error of measurement (SEM), and the minimal detectable change at the 90% confidence level (MDC90), which represent the relative reliability, absolute measurement error, and smallest detectable true change, respectively, were computed for ROM, muscle stiffness, and elasticity, as well as dynamic and isometric torque variables. To adopt parametric tests, visual inspection of histograms and Shapiro–Wilk testing was used to test the normality of the variables while homoscedasticity was assessed and verified through Levene tests. A three-way mixed repeated-measures ANOVA was performed with Time (Pre vs Post) and Protocol (Control, Static Stretching, Stretching + PAPE) as within-subject factors, and Sex (male vs female) as a between-subject factor. Holm–Bonferroni adjustments were applied to all pairwise comparisons within each outcome family for controlling type 1 errors. The relative changes (%Δ) were calculated as the percentage difference between baseline and post-intervention conditions ([(Mean^b^ − Mean^a^)/Mean^a^] × 100). Effect sizes were interpreted as trivial, small, moderate, or large when *d* values were < 0.2, 0.2–0.4, 0.4–0.8, and > 0.8, respectively (Cohen, 1988). Plot-results illustrated descriptive values of main variables split for sex (see Supplementary material). All statistical procedures were performed using SPSS 23.0 (IBM, Chicago, IL, USA) and the level of statistical significance was set at *p* < 0.05.

## Results

Test–retest reliability for all measurements was high. Shoulder ROM demonstrated excellent reliability (ICC = 0.91, SEM = 1.8°, MDC90 = 4.2°). Myoton-derived stiffness and elasticity showed good reliability (ICC = 0.84–0.88, SEM = 6.7–9.1 N·m^−1^, MDC90 = 15.5–21.1 N·m^−1^). Dynamic and isometric torque variables demonstrated excellent consistency (ICC = 0.88–0.95), with SEM values of 3.4–5.8 N·m and MDC90 values of 7.9–13.6 N·m, whereas RFD reliability ranged from ICC = 0.90–0.94 (SEM = 6.2–11.5 N·m·s^−1^, MDC90 = 14.5–26.9 N·m·s^−1^). These indices confirm that changes exceeding these MDC thresholds represent true physiological changes rather than noise, consistent with accepted methodological standards (Weir 2005). All variables met assumptions for parametric analysis (Shapiro–Wilk and Levene tests; all *p* > 0.05).

## Primary outcomes

### Shoulder range of motion (ROM)

There was a significant main effect of Protocol (*p* = 0.007), together with a significant Time × Protocol interaction (*p* = 0.039), indicating that ROM improvements varied depending on the intervention. Holm-Bonferroni-corrected pairwise comparisons showed that SS/PAPE produced greater ROM than CON (*p* = 0.018, d = 0.82) and SS (*p* = 0.041, d = 0.31), while SS exceeded CON (*p* = 0.034, d = 0.59). From baseline to post-intervention, ROM increases were large under SS (∆ = 2.90%; *p* < 0.001; *d* = 1.59) and SS/PAPE (∆ = 3.44%; *p* < 0.001; *d* = 1.80) (Table 1). A Sex effect emerged (*p* = 0.040), with females showing greater absolute ROM across conditions.

**Table 1:** Primary outcomes (n = 14), including mean muscle stiffness parameters obtained in passive conditions and mean force parameters (peak & average torque), obtained during maximal dynamic contractions (5 reps) at 180°/s shoulder extension speed

	Control condition			Static stretching condition			Stretching and PAPE condition			Time effect	Protocol effect	Time × protocol
Shoulder ROM (°)	Base	212.36 ± 6.25 [209.12–216.51]	*p* = 0.125 ∆ = 1.26%	Base	212.50 ± 7.30 [208.93–217.06]	***p* < 0.001** **∆ = 2.90%**	Base	214.21 ± 8.20 [210.39–218.89]	***p* < 0.001** **∆ = 3.44%**	***p* < 0.001**	***p* = 0.007**	***p* = 0.039**
	Post	215.08 ± 7.29 [211.32–219.74]		Post	219.35 ± 7.07 [215.42–223.28]		Post	221.85 ± 9.10 [217.10–227.03]				
Pectoralis Stiffness (Nm^−1^)	Base	230.07 ± 45.11 [207.04–253.13]	*p* = 0.729 ∆ = − 2.96%	Base	233.50 ± 22.42 [217.25–249.74]	*p* = 0.051 ∆ = − 3.12%	Base	224.75 ± 31.26 [212.11–237.38]	*p* = 0.813 ∆ = 0.62%	*p* = 0.472	*p* = 0.723	*p* = 0.078
	Post	226.42 ± 31.61 [209.77–243.08]		Post	227.14 ± 31.31 [209.34–244.94]		Post	227.21 ± 32.42 [207.22–245.06]				
Latissimus Stiffness (Nm^−1^)	Base	243.23 ± 22.18 [203.28–276.64]	*p* = 0.726 ∆ = − 2.61%	Base	266.39 ± 20.25 [232.57–300.20]	*p* = 0.461 ∆ = − 4.15%	Base	261.64 ± 20.71 [222.93–300.35]	*p* = 0.411 ∆ = − 2.66%	*p* = 0.387	*p* = 0.236	*p* = 0.904
	Post	237.03 ± 20.35 [200.52–273.54]		Post	255.79 ± 20.91 [216.16–295.40]		Post	254.86 ± 23.64 [217.09–292.62]				
Average Torque (N)	Base	24.21 ± 8.20 [20.42–27.22]	*p* = 0.129 ∆ = 4.25%	Base	27.17 ± 8.44 [23.46–30.01]	*p* = 0.405 ∆ = − 2.68%	Base	26.02 ± 7.03 [22.94–29.10]	***p* = 0.010** **∆ = 7.92%**	***p* = 0.005**	***p* = 0.048**	***p* = 0.004**
	Post	25.74 ± 7.67 [22.34–27.67]		Post	26.46 ± 6.37 [24.23–28.67]		Post	28.40 ± 6.93 [25.52–30.87]				

Control condition (5 min rowing warm-up); Static stretching condition (5 min rowing warm-up followed by 2 × 30 s pectoralis and latissimus static stretching), and; Stretching and PAPE condition (5 min rowing warm-up, followed by static stretching, followed by 3 × 5 elastic bands pull-over contractions). Note. Bold values indicate statistical significance (p 0.05).

### Passive muscle stiffness

Pectoralis stiffness showed no significant effects of Time, Protocol, or Time × Protocol interaction (all *p* > 0.10), while changes from Baseline to Post-intervention did not reach significance (all *p* > 0.10, *d* < 0.20). Similarly, latissimus dorsi stiffness showed no significant effects (all *p* > 0.10), and no changes were obtained within protocols between Baseline and Post-intervention (Table 1). A trend for Sex effect (*p* = 0.073) indicated higher descriptive values in males (see Supplementary material), but this was not statistically significant.

### Dynamic average torque

Dynamic average torque at 180°·s^−1^ demonstrated significant effects of Time (*p* = 0.005), Protocol (*p* = 0.048), and a Time × Protocol interaction (*p* = 0.004). Holm-Bonferroni-corrected pairwise comparisons confirmed that SS/PAPE produced higher Average Torque than CON (*p* = 0.013, d = 0.36) and SS (*p* = 0.015, d = 0.29), while SS did not differ from CON (*p* = 0.092) (Table 1). Within-condition analyses showed moderate increases from baseline to post-intervention under SS/PAPE (∆ = 7.92%; *p* = 0.010; *d* = 0.34).

## Secondary outcomes

### Passive muscle elasticity

Pectoralis elasticity increased over Time (*p* = 0.007). Post-hoc comparisons showed a significant increase after SS (∆ = 4.52%; *p* = 0.013; d = 0.72), while SS/PAPE showed a non-significant trend (∆ = 3.17%; *p* = 0.060). No significant effects of Protocol (*p* = 0.456) or Time × Protocol interaction (*p* = 0.683) were found. Likewise, latissimus elasticity showed no significant changes (Table 2).

**Table 2:** Secondary outcomes (n = 14), including mean muscle elasticity parameters obtained in passive conditions and mean force parameters obtained during maximal dynamic contractions (5 reps) at 180°/s shoulder extension speed

	Control condition			Static stretching condition			Stretching and PAPE condition			Time effect	Protocol effect	Time × protocol
Pectoralis Elasticity	Base	0.94 ± 0.01 [0.87–1.01]	*p* = 0.327 ∆ = 0.82%	Base	0.96 ± 0.02 [0.90–1.03]	***p* = 0.013** **∆ = 4.52%**	Base	0.97 ± 0.01 [0.88–1.05]	*p* = 0.060 ∆ = 3.17%	***p* = 0.007**	*p* = 0.456	*p* = 0.683
	Post	0.96 ± 0.01 [0.90–1.03]		Post	1.01 ± 0.01 [0.93–1.08]		Post	1.01 ± 0.02 [0.90–1.10]				
Latissimus Elasticity	Base	0.77 ± 0.01 [0.71–0.86]	*p* = 0.451 ∆ = − 1.57%	Base	0.74 ± 0.01 [0.68–0.84]	*p* = 0.585 ∆ = − 1.86%	Base	0.74 ± 0.01 [0.70–0.85]	*p* = 0.341 ∆ = − 1.62%	*p* = 0.508	*p* = 0.242	*p* = 0.998
	Post	0.76 ± 0.01 [0.66–0.82]		Post	0.73 ± 0.01 [0.62–0.79]		Post	0.72 ± 0.01 [0.61–0.76]				
Peak Torque (N)	Base	62.69 ± 23.19 [55.72–72.48]	***p* = 0.013** **∆ = 6.68%**	Base	68.61 ± 20.63 [63.81–77.95]	*p* = 0.747 ∆ = − 1.16%	Base	65.64 ± 22.27 [59.23–77.98]	***p* = 0.020** **∆ = 6.61%**	***p* = 0.005**	*p* = 0.241	***p* = 0.019**
	Post	67.17 ± 22.91 [60.11–77.01]		Post	67.82 ± 15.48 [62.97–74.51]		Post	70.28 ± 20.87 [64.36–80.72]				
Time Peak Torque (s)	Base	0.89 ± 0.27 [0.71–1.05]	*p* = 0.120 ∆ = − 2.67%	Base	0.93 ± 0.17 [0.83–1.02]	*p* = 0.348 ∆ = 1.28%	Base	0.92 ± 0.15 [0.82–1.01]	***p* < 0.001** **∆ = **− **10.42%**	*p* = 0.125	*p* = 0.110	*p* = 0.142
	Post	0.86 ± 0.26 [0.70–1.01]		Post	0.95 ± 0.15 [0.85–1.02]		Post	0.83 ± 0.24 [0.70–0.97]				
dRFD (N·s^−1^)	Base	85.97 ± 79.46 [41.81–130.14]	***p* = 0.021** **∆ = 14.97%**	Base	81.28 ± 49.83 [56.74–105.81]	*p* = 0.405 ∆ = − 5.21%	Base	79.69 ± 48.63 [51.76–103.61]	***p* = 0.004** **∆ = 22.17%**	***p* = 0.026**	*p* = 0.285	***p* = 0.004**
	Post	101.11 ± 94.67 [50.85–151.92]		Post	77.25 ± 42.76 [56.23–99.29]		Post	102.39 ± 76.59 [64.52–140.25]				
Work (J)	Base	134.32 ± 61.93 [110.88–157.41]	*p* = 0.060 ∆ = 5.54%	Base	145.88 ± 53.68 [124.16–167.59]	*p* = 0.594 ∆ = − 1.85%	Base	142.07 ± 53.91 [115.03–169.11]	*p* = 0.081 ∆ = 4.14%	*p* = 0.276	*p* = 0.536	***p* = 0.042**
	Post	142.01 ± 58.61 [119.38–165.65]		Post	142.23 ± 45.75 [123.96–161.07]		Post	148.21 ± 44.67 [127.20–169.21]				
Power (w)	Base	201.49 ± 58.05 [147.54–255.45]	***p* = 0.014** **∆ = 9.18%**	Base	209.02 ± 42.73 [182.52–235.52]	*p* = 0.217 ∆ = − 1.13%	Base	213.74 ± 49.25 [161.65–265.83]	***p* = 0.049** **∆ = 7.44%**	*p* = 0.102	*p* = 0.350	***p* = 0.030**
	Post	221.87 ± 58.57 [160.68–283.05]		Post	206.68 ± 38.31 [184.06–229.31]		Post	230.94 ± 47.25 [174.56–287.33]				

Control condition (5 min rowing warm-up); Static Stretching condition (5 min rowing warm-up followed by 2 × 30 s pectoralis and latissimus static stretching), and; Stretching and PAPE condition (5 min rowing warm-up, followed by static stretching, followed by 3 × 5 elastic bands pull-over contractions) [dRFD: Dynamic rate force development]. Note. Bold values indicate statistical significance (p 0.05)

### Dynamic peak torque, time to dynamic peak torque, and rate of force development (dRFD)

A Time × Protocol interaction (*p* = 0.019) showed that peak torque was greater in SS/PAPE compared with SS (*p* = 0.022, *d* = 0.13) and CON (*p* = 0.038, *d* = 0.14). Torque increased over time in CON (*p* = 0.013) and SS/PAPE (*p* = 0.020) but not in SS. Within-condition analysis showed moderate to large increases only in CON (∆ = 6.68%; *p* = 0.013; *d* = 0.40) and SS/PAPE (∆ = 6.61%; *p* = 0.020; *d* = 0.48). A robust Sex effect was present, with males presenting higher values than females (*p* < 0.001) (see Supplementary material). No significant main effects or interactions were observed in Time to Peak Torque (all *p* > 0.10) (Table 2). However, within-protocol comparison showed a significant moderate to large reduction only in SS/PAPE (∆ = − 10.42%; *p* < 0.001; *d* = 0.45). Regarding dRFD, significant effects were found for Time (*p* = 0.026) and Time × Protocol (*p* = 0.004) (Table 2). SS/PAPE produced higher dRFD than SS (*p* = 0.004), with a large increase from baseline to post-intervention (∆ = 28.48%; *p* = 0.004; *d* = 0.61).

### Work and power

Work showed a significant Time × Protocol interaction (p = 0.042), with SS/PAPE exceeding CON (p = 0.041) (Table 2). Trivial within-protocol increases occurred in CON (∆ = 5.54%; p = 0.009; d = 0.16) and SS/PAPE (∆ = 4.14%; p = 0.040; d = 0.14). Power also showed a significant interaction (p = 0.030), with SS/PAPE outperforming SS (p = 0.027) (Table 2). Males consistently exhibited higher work and power than females (p < 0.01).

### Isometric peak torque, time to isometric peak torque, and isometric RFD (iRFD)

Isometric Peak Torque showed no significant effects for Time or Protocol (all *p* > 0.40), although males produced higher torque at all angles (all *p* < 0.01) (see Supplementary Material). A significant Time × Protocol interaction occurred in Time-to-Peak Torque at 90° (*p* = 0.017), with SS/PAPE producing faster contraction times than CON and SS. Significant Time × Protocol interactions were present in iRFD at 90° (*p* = 0.030) and 35° (*p* = 0.041). SS/PAPE produced higher iRFD than CON at 90° (*p* < 0.001, *d* = 2.47) and 35° (*p* < 0.001, *d* = 2.50), and SS at 90° (*p* = 0.007, *d* = 1.29) and 35° (*p* < 0.001, *d* = 2.58). The within-protocol comparisons showed moderate to large increases in iRFD from Baseline to Post-intervention in SS/PAPE at 90° (∆ = 23.32%; *p* < 0.001; *d* = 1.81) and 35° (∆ = 29.60%; *p* = 0.003; *d* = 1.65); with moderate to large reductions in SS at 150° (∆ = − 25.48%; *p* = 0.010; *d* = 1.14) (Table 3).

**Table 3:** Force parameters (n = 14) obtained during isometric maximal voluntary contraction (MVC) at different shoulder extension angle positions (150°, 90° and 35°) [iRFD: isometric rate force development]

	Control condition			Static stretching condition			Stretching and PAPE condition			Time effect	Protocol effect	Time × protocol
Torque Max (N)												
150°	Base	78.36 ± 29.07 [66.10–90.21]	*p* = 0.621 ∆ = 0.41%	Base	79.78 ± 29.35 [69.81–89.93]	*p* = 0.321 ∆ = − 0.27%	Base	78.29 ± 28.92 [65.29–91.28]	*p* = 0.051 ∆ = 4.56%	*p* = 0.436	*p* = 0.828	*p* = 0.393
	Post	79.04 ± 28.51 [67.82–90.28]		Post	79.57 ± 27.37 [69.23–89.90]		Post	82.03 ± 33.01 [67.03–97.03]				
90°	Base	76.22 ± 29.37 [63.43–87.77]	*p* = 0.217 ∆ = − 1.35%	Base	77.75 ± 26.11 [67.68–87.22]	*p* = 0.487 ∆ = − 0.31%	Base	79.01 ± 28.82 [66.51–91.48]	*p* = 0.105 ∆ = − 2.17%	*p* = 0.501	*p* = 0.140	*p* = 0.803
	Post	75.21 ± 29.79 [60.56–88.22]		Post	77.51 ± 24.99 [67.37–87.23]		Post	77.32 ± 30.29 [61.92–92.71]				
35°	Base	63.05 ± 25.86 [51.87–74.24]	*p* = 0.162 ∆ = 0.52%	Base	66.11 ± 18.92 [58.43–73.78]	*p* = 0.055 ∆ = − 4.38%	Base	66.54 ± 23.42 [56.14–77.62]	*p* = 0.661 ∆ = 0.06%	*p* = 0.449	*p* = 0.187	*p* = 0.348
	Post	63.53 ± 24.85 [52.78–74.29]		Post	63.34 ± 19.49 [54.40–72.27]		Post	66.56 ± 22.34 [55.24–76.99]				
Time to Torque Max (s)												
150°	Base	0.34 ± 0.15 [0.23–0.41]	*p* = 0.078 ∆ = − 11.01%	Base	0.30 ± 0.12 [0.22–0.36]	*p* **=** **0.012** **∆ =** **21.43%**	Base	0.32 ± 0.14 [0.24–0.39]	*p* = 0.052 ∆ = − 9.81%	*p* = 0.605	*p* = 0.321	*p* = 0.231
	Post	0.30 ± 0.12 [0.22–0.37]		Post	0.38 ± 0.16 [0.29–0.46]		Post	0.29 ± 0.08 [0.23–0.33]				
90°	Base	0.37 ± 0.16 [0.29–0.46]	*p* **=** **0.049** **∆** **= −** **19.35%**	Base	0.29 ± 0.10 [0.22–0.34]	*p* = 0.061 ∆ = 14.70%	Base	0.34 ± 0.10 [0.27–0.39]	*p* **=** **0.012** **∆ = −** **36.56%**	*p* = 0.264	*p* = 0.151	*p* **=** **0.017**
	Post	0.31 ± 0.11 [0.25–0.39]		Post	0.34 ± 0.15 [0.25–0.43]		Post	0.25 ± 0.08 [0.20–0.29]				
35°	Base	0.28 ± 0.12 [0.21–0.35]	*p* = 0.069 ∆ = − 12.02%	Base	0.28 ± 0.09 [0.22–0.33]	*p* = 0.102 ∆ = 5.92%	Base	0.27 ± 0.10 [0.20–0.32]	*p* **=** **0.002** **∆ =** **− 35.59%**	*p* **=** **0.050**	*p* = 0.098	*p* = 0.346
	Post	0.25 ± 0.10 [0.20–0.32]		Post	0.30 ± 0.09 [0.24–0.35]		Post	0.20 ± 0.07 [0.15–0.23]				
iRFD (N·s^−1^)												
150°	Base	296.66 ± 76.11 [209.44–386.15]	*p* = 0.804 ∆ = 0.44%	Base	324.27 ± 62.26 [203.78–444.77]	*p* **= 0.010** **∆ = −** **25.48%**	Base	280.94 ± 25.04 [224.39–337.50]	*p* = 0.064 ∆ = 8.21%	*p* = 0.416	*p* = 0.985	*p* = 0.298
	Post	297.96 ± 70.65 [219.98–377.41]		Post	258.42 ± 52.66 [201.56–315.29]		Post	306.06 ± 54.97 [242.77–369.36]				
90°	Base	248.93 ± 30.72 [162.74–318.94]	*p* = 0.498 ∆ = 0.30%	Base	312.31 ± 45.47 [248.27–376.34]	*p* **=** **0.008** **∆ =** **− 14.14%**	Base	261.63 ± 33.98 [203.60–319.67]	*p* **< 0.001** **∆ = 23.32%**	*p* = 0.305	*p* = 0.093	*p* **=** **0.030**
	Post	249.67 ± 30.85 [168.98–320.39]		Post	273.61 ± 52.28 [196.72–350.51]		Post	341.19 ± 51.92 [279.83–402.55]				
35°	Base	266.47 ± 55.47 [181.39–351.56]	*p* = 0.068 ∆ = 8.85%	Base	254.54 ± 93.41 [213.33–295.75]	*p* = 0.092 ∆ = − 15.68%	Base	290.25 ± 97.58 [174.54–391.68]	*p* **= 0.003** **∆ = 29.60%**	*p* = 0.222	*p* = 0.051	*p* **= 0.041**
	Post	292.36 ± 56.29 [195.83–378.26]		Post	220.03 ± 98.04 [133.65–373.73]		Post	412.34 ± 37.77 [245.60–533.08]				

Note. Bold values indicate statistical significance (p 0.05)

## Discussion

This study investigated the acute effects of a short-duration static stretching protocol followed by a resistance-based activation designed to induce post-activation performance enhancement (PAPE) on shoulder range of motion (ROM), passive mechanical properties (stiffness and elasticity) of the pectoralis major and latissimus dorsi, and upper-limb neuromuscular performance in trained swimmers. Overall, the findings indicate that combining static stretching with a dynamic-resisted activity (SS/PAPE) can acutely improve selected performance-related variables such as dynamic rate of force development (dRFD), dynamic average torque, and isometric RFD (iRFD) at specific joint angles, while also producing the largest increase in shoulder ROM. However, results indicate that this protocol may not be sufficient to induce measurable reductions in passive muscle stiffness, at least as assessed under the acute conditions of this study. Our findings partially align with prior work showing that adding exercise after stretching can attenuate stretch-induced reductions in upper-limb strength and power (Gelen et al. 2012; Ghareeb et al. 2017; Mascarin et al. 2015). Nevertheless, the magnitude of these improvements was generally moderate and not universal across variables, while the variability across individuals highlighted that SS/PAPE may benefit some swimmers more than others.

Shoulder ROM increased across all protocols (Table 1), with SS/PAPE producing the largest improvement compared to CON (*p* = 0.018, d = 0.82) and SS (*p* = 0.041, d = 0.31). SS also generated greater ROM gains than CON (*p* = 0.034, d = 0.59), consistent with evidence that short-duration (< 60 s) static stretching reliably enhances joint ROM in the upper limbs (Behm et al. 2023; Reid et al. 2018). Importantly, all ROM changes exceeded the error margin, supporting the validity of the measurements. Our protocol’s 5–10-min latency between the conditioning activity and performance testing falls within the optimal window for PAPE expression (Xu et al. 2025), suggesting that combining stretching with resisted activation can preserve or augment flexibility gains. Collectively, these results indicate that such responses appear to be time-sensitive and highly dependent on warm-up sequencing (Behm et al. 2020). In contrast, muscle stiffness showed minimal responsiveness, and only pectoralis elasticity increased over Time (*p* = 0.007), driven primarily by SS condition (∆ = 4.52%; *p* = 0.013; d = 0.72). The absence of significant stiffness reductions is consistent with studies showing that short-duration stretches (< 60 s) often does not produce detectable changes in passive stiffness (Freitas et al. 2018; Konrad and Tilp 2020). The low responsiveness of the pectoralis major and latissimus dorsi may reflect their mechanical and anatomical complexity, characterized by heterogeneous fiber orientation and variable stiffness regulation across functional tasks (Asayama et al. 2021; Leonardis et al. 2019). Meanwhile, competitive swimmers could display chronic adaptations to repetitive overhead loading (Laudner and Sipes 2009) and may require greater stretching exposure to elicit viscoelastic changes (Lima et al. 2019). Finally, because stiffness changes were non-significant, it is uncertain whether instrument sensitivity contributed to the absence of detectable adaptations. Although the MyotonPRO® is validated, its outputs are influenced by factors such as temperature and probe angle (Kranjc et al. 2025; Lettner et al. 2024). Given the good reliability of our measures, meaningful passive mechanical adaptations may simply require longer or repeated stretching exposures to become detectable, as noted in the lower limbs (Freitas et al. 2018). Overall, these results suggest that the ROM improvements observed are unlikely to reflect changes in passive stiffness. Instead, they align with evidence that short-duration static stretching increases ROM primarily through enhanced stretch tolerance rather than measurable alterations in muscle mechanical properties (Avela et al. 2004; Freitas et al. 2018).

The performance-enhancing effects of the dynamic resistance exercise after the stretching reinforced the proposed rationale underlying our study. The significant Time × Protocol interactions collectively indicated that the SS/PAPE condition produced superior acute responses compared with SS or CON. SS/PAPE elicited the most substantial improvements in force production, with peak torque rising by 6.61% (*p* = 0.020), average torque rising by 7.92% (*p* = 0.010) and dRFD increasing by 28.48% (*p* = 0.004). Within-protocol analyses also demonstrated that peak torque was increased in CON (∆ = 6.68%; *p* = 0.013). These effects were evident in both sexes (e.g., average torque increased from ~ 21.35 ± 4.13 to 23.05 ± 4.53 Nm in women, and from ~ 30.70 ± 6.24 to 33.65 ± 5.07 Nm in men). Moreover, SS/PAPE reduced time-to-peak torque by 10.42% (*p* < 0.001), whereas SS tended to yield longer contraction times (Table 2). Collectively, these results align with previous evidence showing that adding a dynamic or resisted task after stretching can preserve or restore force output compared to reductions often reported after stretching alone (Behm et al. 2020; Blazevich et al. 2018). Mechanistically, these improvements in ROM and torque could likely result from synergistic contributions such as increased muscle compliance via stretching and enhanced muscular responsiveness, such as, fast-twitch fiber recruitment, via PAPE (Blazevich and Babault 2019; Finlay et al. 2021). This is the first study to implement this protocol on a sample of swimmers. Therefore, future empirical evidence in other populations is welcome since individuals with regular exposure to flexibility training, such as swimmers, dancers, or gymnasts, may tolerate longer stretching durations without performance decrements (Lima et al. 2019). In this context, strategies that maintain muscle activation through brief, intense contractile activity may help counteract the post-stretch reductions in force output and also attenuate the effects of long transition periods of relative inactivity following warm-up, thereby supporting the acute responses typically associated with PAPE (Cuenca‐Fernández et al. 2024). In addition, transfer research in swimming pool-settings is mandatory especially in short-course events where repeated maximal efforts (starts, turns, underwater pullouts) occur within the PAPE time window.

The consistent post-intervention Work and Power increases under the SS/PAPE condition suggested favourable neuromechanical adjustments that aligned with our conceptual framework for acute performance optimization in swimmers. The elastic band pull-overs used in the PAPE protocol may have contributed to these effects by providing a stimulus that enhanced neuromuscular efficiency through combined flexibility and high-load input over the full ROM (i.e., increased work) (Barbosa et al. 2020). This practical approach could be especially valuable in upper-body dominant sports and contexts where access to traditional gym equipment is limited, as in swimming competition (Cuenca‐Fernández et al. 2024). In contrast, the lack of improvements in SS supported concerns in the literature regarding the performance-inhibiting potential of isolated static stretching without subsequent reactivation (Behm et al. 2020; Kay and Blazevich 2012; Mizuno et al. 2014). The upper-body PAPE literature supports the effectiveness of task-specific conditioning to enhance subsequent sport-relevant tasks (e.g., sprint, throw, and swim performance), particularly when the conditioning mimics the biomechanical demands of the target action (Finlay et al. 2021). In practical terms, these results reinforce that coaches should not treat stretching and PAPE as mutually exclusive in pre-competition routines. Instead, a short static stretch to augment ROM, immediately followed by a brief, sport-specific high-intensity activation could produce combined benefits: increased mobility with heightened explosive capacity. From a performance standpoint, acute ROM increases could translate to a longer path of action in the pull phase of swimming and more favourable hand entry/exit angles, that, together with the improved muscle force, could translate into potential improvements in stroke length and hydrodynamics (Matthews et al. 2017). However, this remains be tested in real conditions.

In contrast to the dynamic measures, isometric peak torque remained stable across all angles and conditions (see Supplementary material). Nevertheless, the SS/PAPE protocol altered the temporal characteristics of force production with SS/PAPE producing substantially less time-to-peak torque than both CON (*p* = 0.037, d = 0.62) and SS (*p* = 0.021, d = 0.74). Within-protocol comparisons further showed that SS/PAPE consistently showed moderate to large decreases in time-to-peak torque from baseline to post-intervention at 90° (∆ =  − 36.60%; *p* = 0.012; d = 0.99), and 35° (∆ =  − 35.59%; *p* = 0.002; d = 0.81). These temporal improvements were accompanied by corresponding gains in iRFD, evidenced by significant Time × Protocol interactions at both 90° (*p* = 0.030) and 35° (*p* = 0.041). SS/PAPE produced markedly greater iRFD than CON (*p* < 0.001, d = 2.47–2.50) and SS (*p* = 0.007 to < 0.001, d = 1.29–2.58) at each of these angles. Within-condition analyses confirmed moderate to large increases in iRFD under SS/PAPE at 90° (∆ = 23.32%; *p* < 0.001; d = 1.81), and 35° (∆ = 29.60%; *p* = 0.003; d = 1.65), while SS consistently produced reductions and CON showed minimal change. Given the significant increases in iRFD at SS/PAPE, these improvements likely reflect enhanced neuromuscular readiness associated with the conditioning activity, such as improved motor unit recruitment (Moriarty et al. 2022). In addition, results indicate a more efficient recruitment of high-threshold motor units at joint angles where swimmers generate propulsive force (i.e., 90° and 35°) (Kasprisin and Grabiner 2000; Chollet et al. 2000). Although a consistent sex effect emerged (*p* = 0.004), with males outperforming females across all angles, the facilitatory impact of SS/PAPE on iRFD was comparable across sexes (see Supplementary material). Overall, while isometric peak torque itself was unaffected, the improved iRFD and reduced time-to-peak torque under SS/PAPE indicate more rapid force initiation, an attribute highly relevant to the mid- and late-pull phases of swimming where propulsive efficiency depends on rapid force transmission.

A significant Sex effect showed that females exhibited greater absolute ROM across all conditions (*p* = 0.040). Meanwhile, absolute male advantage in torque, time to peak and iRFD metrics were large (see Supplementary material). Therefore, although both sexes benefited from the combined protocol in relative terms this may warrant further investigation in studies with larger sample sizes to explore potential sex-based or protocol-specific neuromuscular response differences. For example, future studies should test if female athletes with greater baseline ROM may require less mobility-focused warm-up and more activation emphasis, whereas male athletes with higher baseline stiffness may benefit from additional eccentric or soft-tissue preparatory work, especially during re-warm-up periods (Cuenca‐Fernández et al. 2024). On the other hand, although SS/PAPE produced moderate improvements in RFD and dynamic torque, the practical translation to competitive performance remains uncertain since dryland effects do not necessarily equate to measurable differences in stroke mechanics or lap times (Cuenca-Fernández et al. 2022). In any case, there is evidence that upper-body strength and power are relevant for sprint swimming performance. For example, a recent study found moderate to strong negative correlations between upper-limb isokinetic strength and 25 m sprint times in elite and sub-elite swimmers, indicating that greater torques were associated with faster swim times (Carvalho et al. 2023). Similarly, improvements in neuromuscular power (e.g., arm pull force, RFD) have been pointed out as more relevant than work output per se in contributing to swim propulsion, stroke rate, and velocity, particularly in sprint events (Gatta et al. 2015). At last, given that even small improvements in start or turn times (on the order of 1–2%) can influence final race rankings in sprints (Parrott 2023), recent analyses show that a 1% improvement in block start time in 50 m freestyle might shift a swimmer from fourth to second place (Arellano et al. 2022). The magnitude of the significant changes observed in this study provides useful context for swimming performance, where outcomes are often decided within 0.05–0.20 s. For example, the 7–8% increase in dynamic torque and ~ 20 to 30% improvement in RFD under SS/PAPE represent faster force initiation and greater early-phase force production, which reflect qualities relevant to the catch and mid-pull phases of freestyle and butterfly (Chollet et al. 2000), where rapid force transmission contributes to propulsion. Similarly, reducing time-to-peak torque by 10–35% may assist swimmers during explosive actions such as starts and turns, where rapid upper-limb force application influences block clearance, underwater pullouts, and wall transition times (Parrott 2023). Meanwhile, the observed 3–4% increases in shoulder ROM, while modest, could theoretically facilitate more favourable entry angles or slightly longer stroke paths, both of which have been associated with improvements in stroke efficiency (Matthews et al. 2017). These considerations remain conceptual, but they offer practical insight into how the combined SS/PAPE protocol may influence race-relevant components even if direct in-water effects were not measured.

Apart from this, future studies should: (1) explicitly test sex-specific protocols and dosing (stretch duration, number of activation reps/sets, and recovery intervals) to identify optimal combinations for different events (sprint vs. middle-distance) and athlete profiles; (2) Investigate chronic adaptations to regular inclusion of SS/PAPE in training cycles to assess if acute potentiation and mobility gains translate into long-term changes in stiffness, architecture, or performance, and; (3) Use multimodal assessment (Shear wave elastography, MyotonPRO®, ultrasound fascicle imaging, and transcranial magnetic stimulation) to disentangle structural versus neural contributions to acute and chronic responses (Lettner et al. 2024). Several limitations should be acknowledged. First, all performance testing was conducted in a dryland laboratory setting; therefore, the observed improvements in in dynamic torque (Δ ≈ 7.9%) and dRFD (Δ ≈ 22%) cannot be directly extrapolated to in-water race performance or translated into specific time reductions. Second, the sample size (n = 14) limited statistical power and precluded robust sex-specific subgroup analyses, making these exploratory only. Third, although MyotonPRO® provides reliable estimates of passive mechanical properties, it may be insufficiently sensitive to detect rapid or subtle acute changes that can be affected by factors such as temperature and physiological interactions, as well as probe angle (Lettner et al. 2024; Kranjc et al. 2025), and the present non-significant stiffness outcomes cannot rule out measurement insensitivity as a contributing factor. Fourth, the single post-intervention timepoint after the aerobic warm-up (~ 10 min) may have overlooked very short-term or delayed responses. Finally, participant characteristics (trained adolescent/young-adult swimmers) restrict generalisability to elite and youth categories. These limitations should be considered when interpreting the present findings and highlight the need for future studies incorporating in-water assessments, larger and sex-balanced samples, multi-timepoint testing, and additional mechanical assessment tools.

## Conclusion

This study showed that combining short-duration static stretching with a brief resisted activation (SS/PAPE) in the upper limbs produced the greatest acute increase in shoulder ROM and moderate improvements in dynamic average and peak torque, compared with stretching alone or control. Changes in passive muscle stiffness and elasticity of the pectoralis major and latissimus dorsi were small and mostly non-significant, indicating that the observed ROM improvements are likely related to enhanced stretch tolerance rather than measurable alterations in tissue mechanical properties. Within this laboratory context, the combined protocol of 5 min of general aerobic activation (e.g., rowing), followed by 2 × 30 s static stretches of the latissimus dorsi and pectoralis major, and then 3 × 5 resisted band pull-overs at a high but submaximal intensity (≈RIR 1–2), appeared to support short-term performance enhancements of the upper limbs within ~ 10 min after the aerobic activation, although responses varied across individuals and were not consistently superior across all outcomes. Importantly, these findings are limited to dryland assessments, and no inference can be made regarding effects on in-water performance or race outcomes. Therefore, any practical application of this approach should remain exploratory until verified in sport-specific settings. Future research should examine its transfer to competitive swimming tasks, investigate long-term adaptations, and determine whether protocol adjustments or athlete characteristics influence the magnitude and relevance of the acute responses observed here.

## Supplementary Information

Below is the link to the electronic supplementary material.

Supplementary file1 (PDF 357 KB)

## Abbreviations

CON: Control
MVC: Maximal voluntary contraction
PAP: Post-activation potentiation
PAPE: Post-activation performance enhancement
ROM: Range of motion
RFD: Rate of force development
RIR: Reps in reserve
SS: Static stretching
