# Supplementary material for: Stretching and potentiation for performance optimization: effects on upper limbs in competitive swimmers
Source: Eur J Appl Physiol. 2026 Jan 16;126(6):3071–86. doi: 10.1007/s00421-026-06133-9 (PMC13287152; doi:10.1007/s00421-026-06133-9)
Supplement: Supplementary file 1 — Supplementary file1 (PDF 357 KB) [file 421_2026_6133_MOESM1_ESM.pdf]

Supplementary Material: Isometric force parameters (n = 14), obtained during maximal voluntary contraction (MVC) at different shoulder extension angle positions (150º, 90º and 35º) [iRFD: Isometric rate force development].

		CONTROL CONDITION			STATIC STRETCHING CONDITION			STRETCHING and PAPE CONDITION			Time effect	Protocol effect	Time × Protocol
**Torque Max (N)**	**150º**	Base	78.36 ± 29.07 [66.10 – 90.21]	p = 0.621 Δ = 0.41%	Base	79.78 ± 29.35 [69.81 – 89.93]	p = 0.321 Δ = -0.27%	Base	78.29 ± 28.92 [65.29 – 91.28]	p = 0.051 Δ = 4.56%	p = 0.436	p = 0.828	p = 0.393
		Post	79.04 ± 28.51 [67.82 – 90.28]		Post	79.57 ± 27.37 [69.23 – 89.90]		Post	82.03 ± 33.01 [67.03 – 97.03]				
	**90º**	Base	76.22 ± 29.37 [63.43 – 87.77]	p = 0.217 Δ = -1.35%	Base	77.75 ± 26.11 [67.68 – 87.22]	p = 0.487 Δ = -0.31%	Base	79.01 ± 28.82 [66.51 – 91.48]	p = 0.105 Δ = -2.17%	p = 0.501	p = 0.140	p = 0.803
		Post	75.21 ± 29.79 [60.56 – 88.22]		Post	77.51 ± 24.99 [67.37 – 87.23]		Post	77.32 ± 30.29 [61.92 – 92.71]				
	**35º**	Base	63.05 ± 25.86 [51.87 – 74.24]	p = 0.162 Δ = 0.52%	Base	66.11 ± 18.92 [58.43 – 73.78]	p = 0.055 Δ = -4.38%	Base	66.54 ± 23.42 [56.14 – 77.62]	p = 0.661 Δ = 0.06%	p = 0.449	p = 0.187	p = 0.348
		Post	63.53 ± 24.85 [52.78 – 74.29]		Post	63.34 ± 19.49 [54.40 – 72.27]		Post	66.56 ± 22.34 [55.24 – 76.99]				
**Time to Torque Max (s)**	**150º**	Base	0.34 ± 0.15 [0.23 – 0.41]	p = 0.078 Δ= -11.01%	Base	0.30 ± 0.12 [0.22 – 0.36]	**p = 0.012 Δ = 21.43%**	Base	0.32 ± 0.14 [0.24 – 0.39]	p = 0.052 Δ = -9.81%	p = 0.605	p = 0.321	p = 0.231
		Post	0.30 ± 0.12 [0.22 – 0.37]		Post	0.38 ± 0.16 [0.29 – 0.46]		Post	0.29 ± 0.08 [0.23 – 0.33]				
	**90º**	Base	0.37 ± 0.16 [0.29 – 0.46]	**p = 0.049 Δ= -19.35%**	Base	0.29 ± 0.10 [0.22 – 0.34]	p = 0.061 Δ = 14.70%	Base	0.34 ± 0.10 [0.27 – 0.39]	**p = 0.012 Δ = -36.56%**	p = 0.264	p = 0.151	**p = 0.017**
		Post	0.31 ± 0.11 [0.25 – 0.39]		Post	0.34 ± 0.15 [0.25 – 0.43]		Post	0.25 ± 0.08 [0.20 – 0.29]				
	**35º**	Base	0.28 ± 0.12 [0.21 – 0.35]	p = 0.069 Δ= - 12.02%	Base	0.28 ± 0.09 [0.22 – 0.33]	p = 0.102 Δ = 5.92%	Base	0.27 ± 0.10 [0.20 – 0.32]	**p = 0.002 Δ = -35.59%**	**p = 0.050**	p = 0.098	p = 0.346
		Post	0.25 ± 0.10 [0.20 – 0.32]		Post	0.30 ± 0.09 [0.24 – 0.35]		Post	0.20 ± 0.07 [0.15 – 0.23]				
**iRFD ($N \cdot s^{-1}$)**	**150º**	Base	296.66 ± 76.11 [209.44 – 386.15]	p = 0.804 Δ = 0.44%	Base	324.27 ± 62.26 [203.78 – 444.77]	**p = 0.010 Δ = -25.48%**	Base	280.94 ± 25.04 [224.39 – 337.50]	p = 0.064 Δ = 8.21%	p = 0.416	p = 0.985	p = 0.298
		Post	297.96 ± 70.65 [219.98 – 377.41]		Post	258.42 ± 52.66 [201.56 – 315.29]		Post	306.06 ± 54.97 [242.77 – 369.36]				
	**90º**	Base	248.93 ± 30.72 [162.74 – 318.94]	p = 0.498 Δ = 0.30%	Base	312.31 ± 45.47 [248.27 – 376.34]	**p = 0.008 Δ = -14.14%**	Base	261.63 ± 33.98 [203.60 – 319.67]	**p < 0.001 Δ = 23.32%**	p = 0.305	p = 0.093	**p = 0.030**
		Post	249.67 ± 30.85 [168.98 – 320.39]		Post	273.61 ± 52.28 [196.72 – 350.51]		Post	341.19 ± 51.92 [279.83 – 402.55]				
	**35º**	Base	266.47 ± 55.47 [181.39 – 351.56]	p = 0.068 Δ = 8.85%	Base	254.54 ± 93.41 [213.33 – 295.75]	p = 0.092 Δ = -15.68%	Base	290.25 ± 97.58 [174.54 – 391.68]	**p = 0.003 Δ = 29.60%**	p = 0.222	p = 0.051	**p = 0.041**
		Post	292.36 ± 56.29 [195.83 – 378.26]		Post	220.03 ± 98.04 [133.65 – 373.73]		Post	412.34 ± 37.77 [245.60 – 533.08]				

Plot A. Mean ± SD (Standard deviation) shoulder range of motion values collected separately in women and men. (* = Sex effect; p = 0.040)

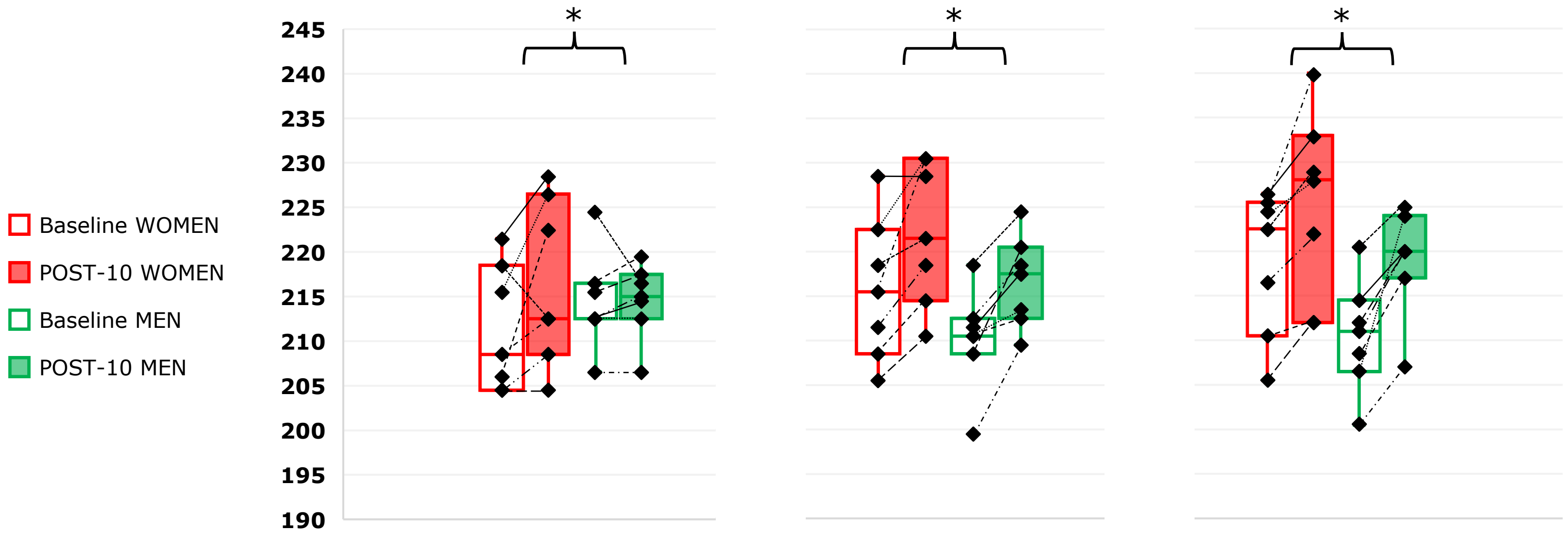

Shoulder Range of Motion (º)		CONTROL CONDITION	STATIC STRETCHING CONDITION	STRETCHING + PAPE CONDITION
WOMEN	Baseline	211.28 ± 7.09	215.78 ± 8.07	218.78 ± 8.17
	POST-10 min	216.50 ± 9.30	222.07 ± 8.03	225.14 ± 9.49
MEN	Baseline	214.35 ± 5.49	210.21 ± 5.67	210.50 ± 6.30
	POST-10 min	214.57 ± 4.20	216.64 ± 5.14	219.01 ± 5.94

Plot B. Mean ± SD (Standard deviation) pectoralis stiffness values collected separately in women and men.

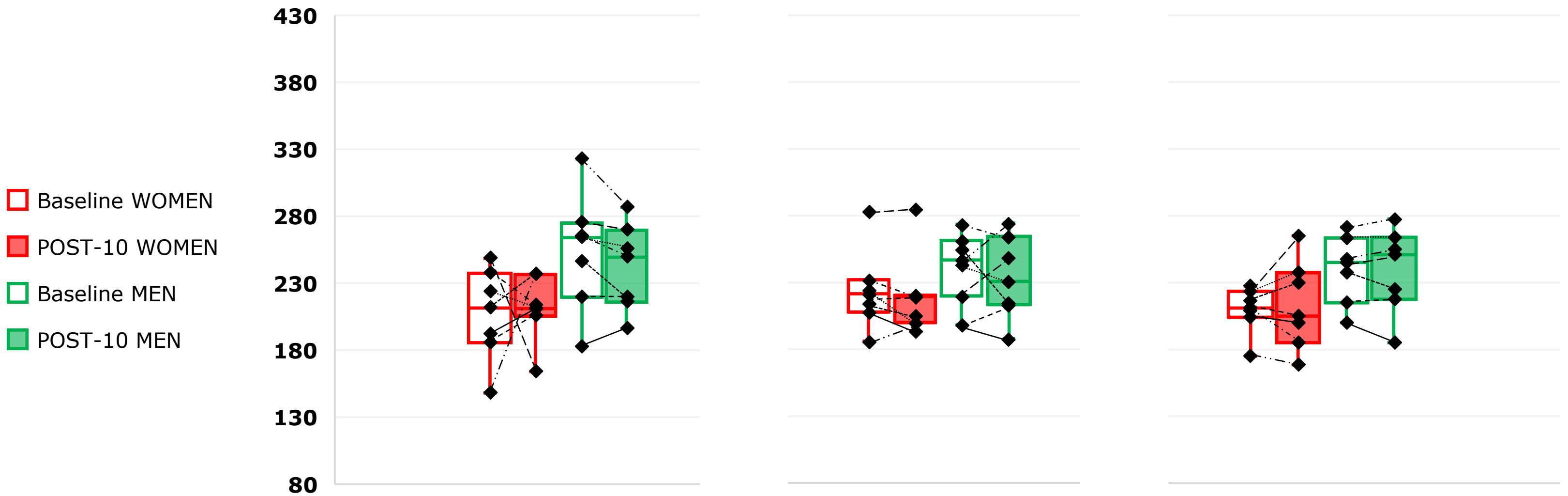

Pectoralis Stiffness (N/m²)		CONTROL CONDITION	STATIC STRETCHING CONDITION	STRETCHING + PAPE CONDITION
**WOMEN**	**Baseline**	206.64 ± 34.43	224.29 ± 30.00	209.50 ± 17.28
	**POST-10 min**	211.07 ± 24.31	220.64 ± 30.30	213.00 ± 33.14
**MEN**	**Baseline**	253.50 ± 44.19	242.71 ± 25.63	240.01 ± 25.35
	**POST-10 min**	241.79 ± 32.33	233.64 ± 30.82	239.29 ± 31.84

Plot C. Mean ± SD (Standard deviation) latissimus dorsi values collected separately in women and men.

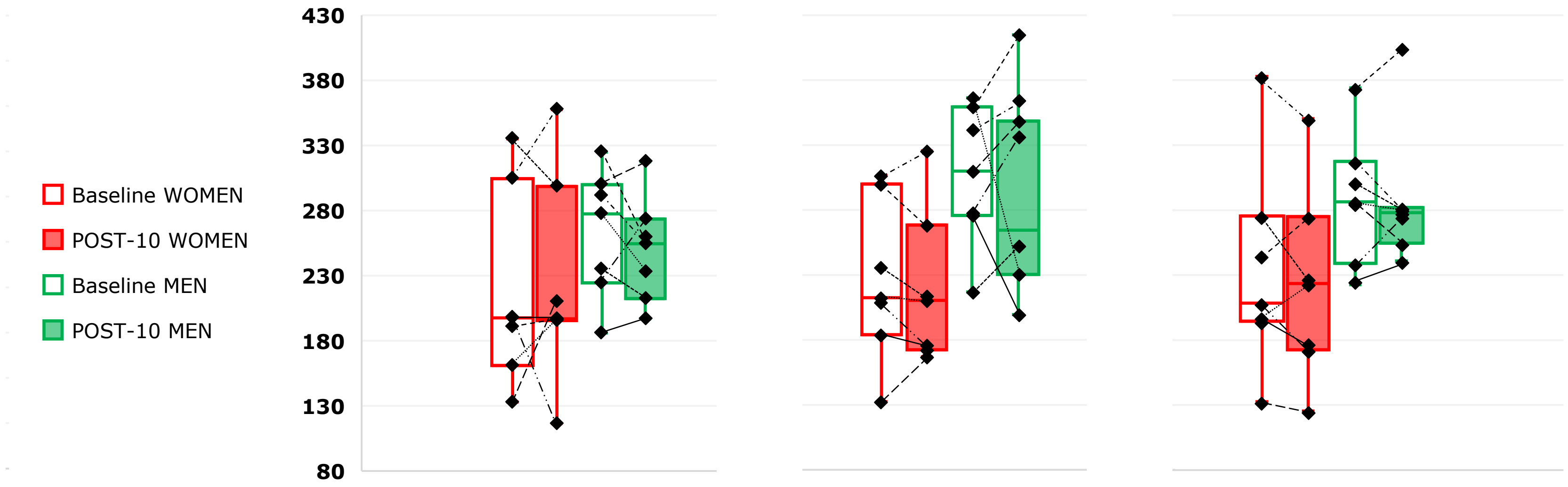

Latissimus Dorsi Stiffness (N/m²)		CONTROL CONDITION	STATIC STRETCHING CONDITION	STRETCHING + PAPE CONDITION
**WOMEN**	**Baseline**	217.14 ± 74.73	225.79 ± 61.96	233.79 ± 79.47
	**POST-10 min**	224.43 ± 79.00	219.14 ± 58.60	221.71 ± 74.27
**MEN**	**Baseline**	262.79 ± 49.04	307.00 ± 53.90	289.50 ± 50.22
	**POST-10 min**	249.64 ± 40.25	292.43 ± 76.33	288.00 ± 53.82

Plot D. Mean ± SD (Standard deviation) shoulder peak torque values collected separately in women and men during maximal dynamic contractions (5 reps) at 180º/s shoulder extension speed. (# = Sex effect; p = 0.002)

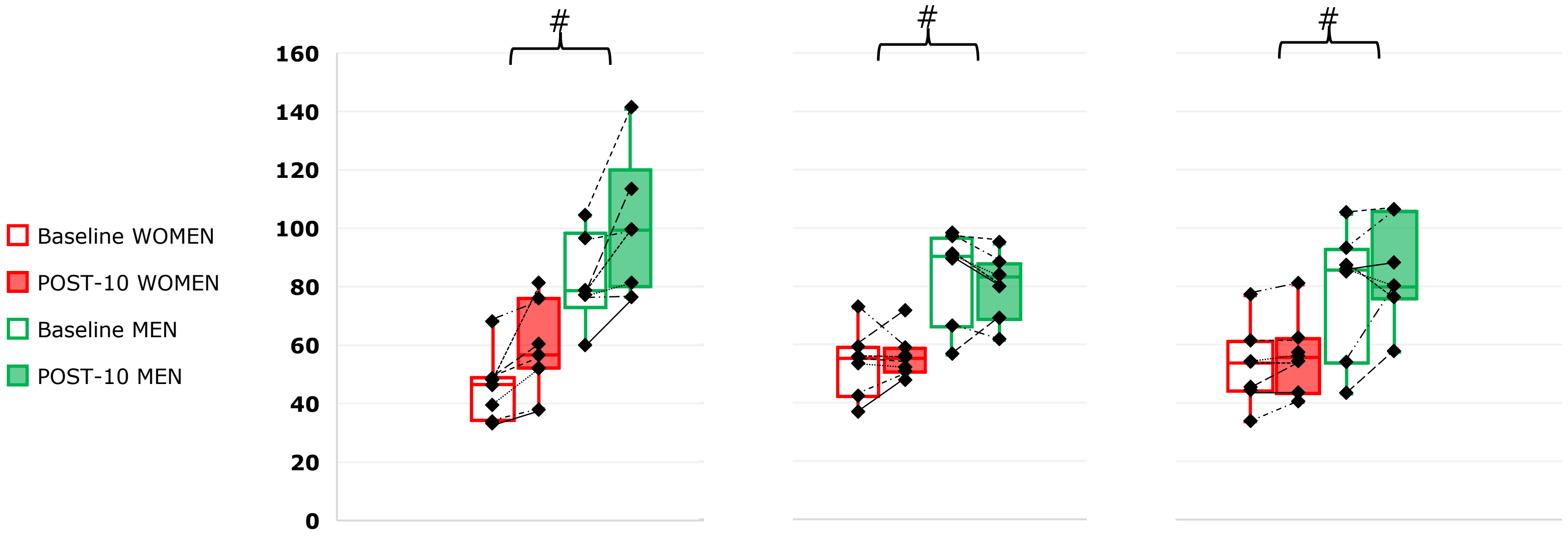

Peak Torque (N/m)		CONTROL CONDITION	STATIC STRETCHING CONDITION	STRETCHING + PAPE CONDITION
**WOMEN**	**Baseline**	45.67 ± 11.81	53.49 ± 11.60	52.59 ± 13.84
	**POST-10 min**	59.92 ± 14.77	55.89 ± 7.72	56.05 ± 13.27
**MEN**	**Baseline**	82.55 ± 15.64	83.73 ± 15.95	78.69 ± 22.02
	**POST-10 min**	101.69 ± 23.34	79.76 ± 11.28	84.52 ± 17.15

Plot E. Mean ± SD (Standard deviation) shoulder average torque values collected separately in women and men during maximal dynamic contractions (5 reps) at 180º/s shoulder extension speed. (# = Sex effect; p = 0.001)

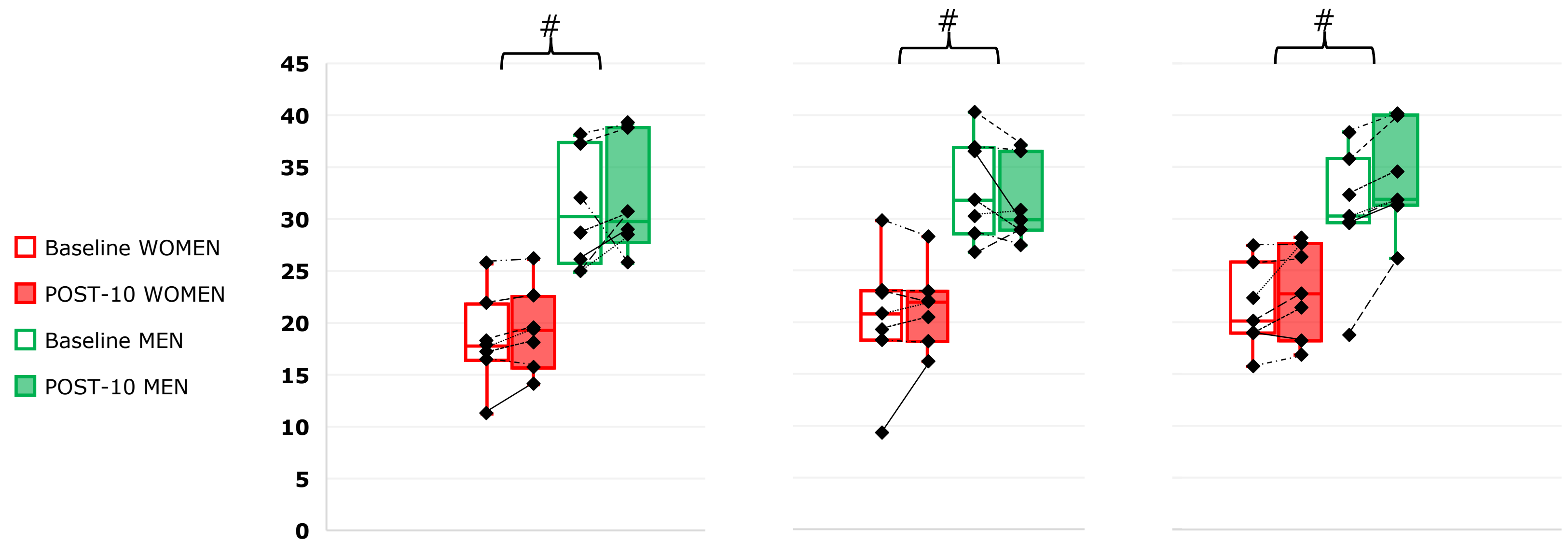

Average Torque (N/m)		CONTROL CONDITION	STATIC STRETCHING CONDITION	STRETCHING + PAPE CONDITION
**WOMEN**	**Baseline**	18.32 ± 4.52	20.48 ± 6.20	21.35 ± 4.13
	**POST-10 min**	19.30 ± 4.07	21.43 ± 3.84	23.05 ± 4.53
**MEN**	**Baseline**	31.30 ± 5.59	32.99 ± 4.97	30.70 ± 6.24
	**POST-10 min**	31.91 ± 5.66	31.48 ± 3.77	33.65 ± 5.07

Plot F. Mean ± SD (Standard deviation) iRFD descriptive values collected separately in women & men at 150, 90 and 35º shoulder extension (# = Sex effect; p = 0.004).

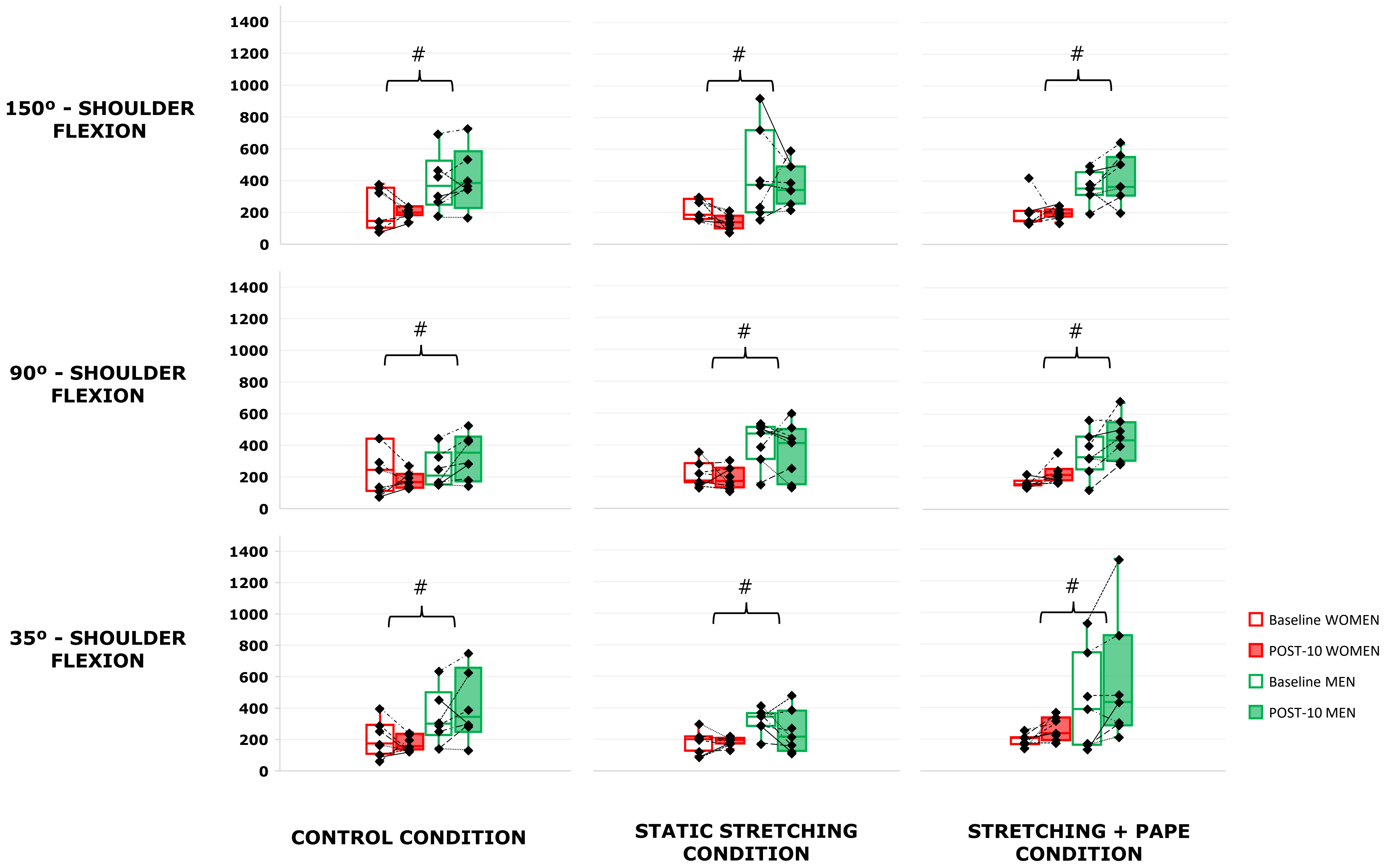

Plot F. Mean ± SD (Standard deviation) iRFD descriptive values collected separately in women & men at 150, 90 and 35º shoulder extension (# = Sex effect; p = 0.004).

CONTROL CONDITION

RFD ($N \cdot s^{-1}$)	WOMEN		MEN	
	**Baseline**	**POST-10 min**	**Baseline**	**POST-10 min**
**150º**	214.94 ± 32.54	249.72 ± 51.08	392.00 ± 81.92	248.00 ± 66.71
**90º**	249.72 ± 51.08	179.70 ± 52.61	248.00 ± 16.71	331.30 ± 51.39
**35º**	209.81± 41.22	177.54 ± 49.76	349.52 ± 72.45	414.64 ± 30.59

STATIC STRETCHING CONDITION

RFD ($N \cdot s^{-1}$)	WOMEN		MEN	
	**Baseline**	**POST-10 min**	**Baseline**	**POST-10 min**
**150º**	219.46 ± 61.60	142.37 ± 47.27	429.09 ± 286.09	374.48 ± 129.76
**90º**	215.95 ± 77.83	192.45 ± 68.07	408.67 ± 134.65	354.79 ± 173.89
**35º**	192.81± 65.46	189.25 ± 27.87	316.27± 75.71	250.82 ±133.64

STRETCHING + PAPE CONDITION

RFD ($N \cdot s^{-1}$)	WOMEN		MEN	
	**Baseline**	**POST-10 min**	**Baseline**	**POST-10 min**
**150º**	200.74 ± 98.74	195.73 ± 33.95	361.16 ± 95.47	416.40 ± 149.92
**90º**	171.32 ± 28.51	232.04 ± 63.39	351.95 ± 138.03	450.35 ± 134.88
**35º**	197.04 ± 36.83	265.64 ± 75.63	431.11 ± 131.16	559.20 ± 140.72
